# Virus-induced gene silencing in *Dianthus* by an apple latent spherical virus vector system

**DOI:** 10.5511/plantbiotechnology.25.0922a

**Published:** 2026-03-25

**Authors:** Koji Tanase, Ichiro Kasajima, Nobuyuki Yoshikawa

**Affiliations:** 1 Institute of Vegetable and Floriculture Science, National Agriculture and Food Research Organization (NARO), Tsukuba, Ibaraki 305-0852, Japan; 2 Faculty of Agriculture, Iwate University, Morioka, Iwate 020-8550, Japan; 3 Nishinihon Station, Center for Seeds and Seedlings, National Agriculture and Food Research Organization (NARO), Kasaoka, Okayama 714-0054, Japan

**Keywords:** ALSV, carnation, *Dianthus*, VIGS, virus vector

## Abstract

For the functional analysis of *Dianthus* and carnation endogenous genes, we investigated a viral vector derived from the apple latent spherical virus (ALSV) as a tool for reverse genetic analysis. ALSV can infect the aerial parts, such as leaves and flower organs, of *Dianthus* and carnation plants, without causing viral symptoms. Partial sequences of the *chalcone synthase* (*CHS*), *1-aminocyclopropane-1-carboxylate* (*ACC*) *synthase* (*ACS*), and *ACC oxidase* (*ACO*) genes were cloned into the ALSV vector and then used to infect the plant. Plants infected with ALSV vectors carrying these genes exhibited knockdown phenotypes typical of *CHS*, *ACS*, and *ACO*. Plants infected with the ALSV vector carrying *CHS* showed white flower petals, whereas those infected with the ALSV vector carrying *ACS* and *ACO* generated long-lived flowers. Thus, ALSV vectors can promote virus-induced gene silencing (VIGS) in the petals and gynoecium. ALSV infects plants without viral symptoms and effectively induces VIGS in several flower organs; thus, the ALSV vector is a valuable tool for determining the functions of genes of interest in *Dianthus* and carnation plants.

## Introduction

*Dianthus*, a genus with approximately 340 species, is widely distributed in southern Europe and Asia, from the Mediterranean coast to Japan (Hamilton and Walters 1989). Many species of *Dianthus* are important horticultural crops, including carnations (*Dianthus caryophyllus*), *D. chinensis*, and *D. barbatus*, which are used as cut flowers, pot plants, and garden plants. Particularly, carnations are among the most produced floricultural crops worldwide.

The breeding, cultivation, and genetic modification of *Dianthus* have been studied. The genome sequence of the carnation cultivar ‘Francesco’ was determined using next-generation sequencing data (Yagi et al. 2014) released from the Carnation DB (https://carnation.kazusa.or.jp/). Several genes of interest have been identified using this database and relevant next-generation sequencing data, such as genes involved in petal color and flower senescence (Ohmiya et al. 2013, 2014; Tanase et al. 2012, 2015). However, further detailed analyses of the functions of the genes obtained and those to be identified in the future are needed. Reverse genetics, an experimental molecular genetics technique for modifying genes and analyzing the resulting phenotype, is often used to study the function of endogenous genes in plants. Reverse genetics studies have used transgenic plants with knockdown or knockout genes, using techniques such as T-DNA insertion mutagenesis, genome editing, and gene silencing. Gene silencing is one of the most extensively applied techniques for the functional characterization of different target genes in many plant species, including horticultural crops (Pandey et al. 2015). RNA interference, based on the generation of transgenic plants in which DNA is modified using genetic engineering techniques, is the most commonly used gene silencing technology.

Transgenic carnation plants have been generated in recent years. For example, a transgenic carnation with improved flower life was transformed with the *1-aminocyclopropane-1-carboxylate* (*ACC*) *oxidase* (*ACO*) gene in an antisense orientation (Savin et al. 1995). Transgenic blue carnations with delphinidin accumulation were produced by introducing a *flavonoid 3′ 5′-hydroxylase* (*F3′ 5′H*) gene from pansy (Tanaka et al. 1998). *Agrobacterium*-mediated genetic transformation methods have been used for carnations; however, they exhibited very low transformation efficiency (Nontaswatsri et al. 2002, 2004). In addition, the transformation efficiency varied greatly among varieties. Viral vector technology is used to infect plants with a plant virus carrying a foreign gene. This technology can suppress gene expression through virus-induced gene silencing (VIGS), or produce high levels of target protein expression in the plant body by exploiting the ability of the virus to replicate in plants. As this technology can produce transformants more easily and quickly than *Agrobacterium*-mediated transformation, it is used to study gene function in plants, including floricultural crops (Liu et al. 2021; Tanase et al. 2019; Yamagishi and Yoshikawa 2010).

Apple latent spherical virus (ALSV) is a non-pathogenic virus isolated from an apple tree (Nakamura et al. 2011). ALSV has only been detected in a single apple tree, excluding experimentally infected plants, and has advantages as a viral vector. It has a wide host range, latently infects most plant species without viral symptoms, and uniformly infects the upper parts of plants (Igarashi et al. 2009; Kasajima et al. 2017; Yamagishi and Yoshikawa 2009; Yamagishi et al. 2011). The present study aimed to investigate ALSV as a novel high-throughput method for estimating the functions of *Dianthus* genes. To achieve this, we inoculated ALSV vectors into *Dianthus* cultivars.

## Materials and methods

### Plant materials

Two *Dianthus* cultivars, *Dianthus chinensis* × *barbatus* ‘Telstar Picotee’ and ‘Telstar Crimson’ (Takii Seeds, Kyoto, Japan), and a carnation cultivar, *Dianthus caryophyllus* L. ‘Ariel’ (Japan Agribio Company Limited, Shizuoka, Japan), were used in this study. Seeds of *Dianthus* cultivars were stored at 4°C and were sown in plug trays on soil mix (Sakata Seed Co., Kanagawa, Japan). The germinated seedlings used for inoculation experiments were grown under fluorescent lights at 200 µmol m^−2^ s^−1^ for 16 h of light and 8 h of dark cycles, at 23°C. After 7 days, the inoculated plants were transplanted into 9 cm pots and used for further experiments. Carnation plants were used for viral inoculation in vitro. Carnation plant shoots (5 cm in length) were collected from plants grown in a greenhouse under a natural photoperiod. The surface of the shoots was sterilized for 15 min in 1% sodium hypochlorite and one drop of Tween-20, followed by four rinses in sterile distilled water. Leaves were carefully removed from the nodes and the apical meristems were cut into 5 mm explants. Single nodal plants were cultured on Murashige and Skoog (MS) medium containing 20 g l^−1^ and 2 g l^−1^ Gellan Gum for one month (Nontaswatsri et al. 2004) under florescent grow lights at 100 µmol m^−2^ s^−1^ and a 16 h light and 8 h dark cycle, at 23°C. The in vitro plants were used for inoculation experiments. For the acclimatization of in vitro plants, unvitrified shoots with developed roots were transferred to the soil mix in plug trays with maintained humidity. After hardening, plants were transplanted to 9 cm pots and grown under fluorescent grow lights at 200 µmol m^−2^ s^−1^ and 16 h light and 8 h dark cycle, at 23°C.

For measurement of flower longevity, lines were grown in a containment greenhouse. Flowers were tagged at the flower-opening stage (day 0), that is, when the outer petals became orientated at right angles to the stem. The flower longevity of each line was determined as the number of days from day 0 until the flowers lost their flower quality, which was defined as flower wilting with inrolling, browning of the petal edge without inrolling, or desiccating without inrolling. Flowers (*n*=6) were evaluated daily. A significant difference (*p*<0.05) was found by two-sample *t*-test using ‘Telstar Picotee’ line No. 1 infected with wt-ALSV (Supplementary Tables S2, S3) as the control.

### RNA extraction, cloning, and plasmid construction

Plant organs for RNA extraction were frozen using liquid nitrogen and stored at −80°C until use. Total RNA was extracted from plant organs using TRIzol reagent according to the manufacturer’s protocol.

Full-length cDNA of *DsCHS1* was generated via PCR using Ex Taq (TaKaRa Bio Inc., Shiga, Japan) and a forward primer (DsCHSFullfw) and a reverse primer (DsCHSFullrv) (Supplementary Table S1) under the following reactions: 30 cycles of 30 s at 94°C, 20 s at 57°C, and 2 min at 72°C. The fragment was cloned using the pGEM-T Easy Vector System (Promega Corporation, Madison, WI, USA). Clone sequences were determined by dideoxy chain termination using an Applied Biosystems Genetic Analyzer 3500 (Thermo Fisher Scientific K.K., Waltham, MA, USA).

**Figure 1:**
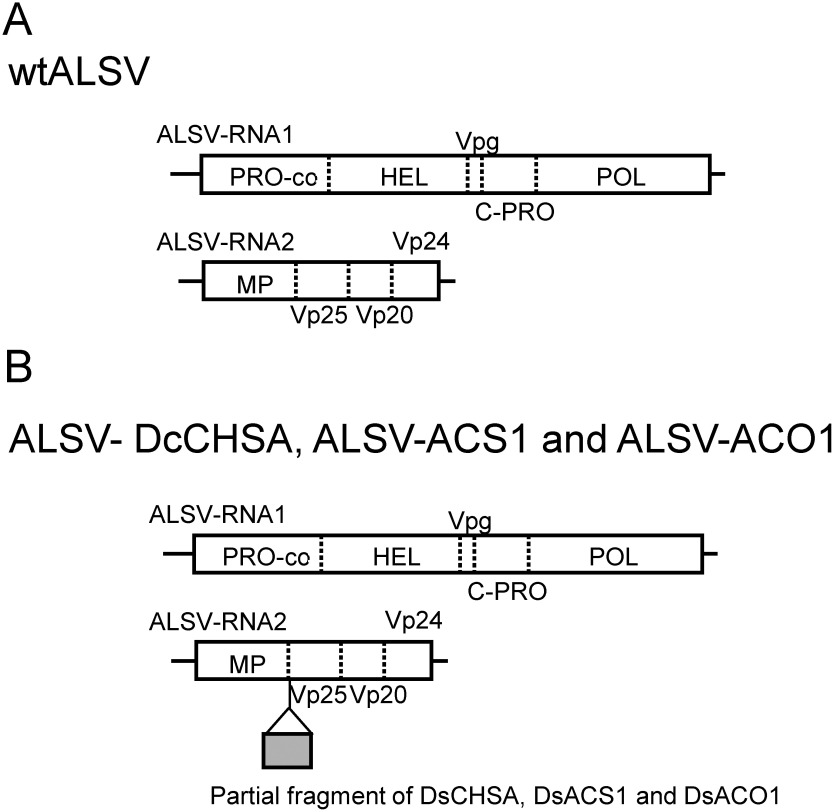
Figure 1. Structure of the ALSV vector. (A) ALSV-RNA1 genome encoding protease cofactor (PRO-co), NTP-binding helicase (HEL), viral protein genome-linked (Vpg), cysteine protease (C-PRO), and RNA polymerase (POL). ALSV-RNA2 genome encoding movement protein (MP) and capsid protein Vp25, Vp20, and Vp24. (B) Cloning site is inserted in the plasmid clone of the RNA2 genome between MP and Vp25.

Vectors containing each target fragment of 201 bp DNA were created and cloned into the pEALSR2L5R plasmid. The 201 bp DNA fragments corresponding to each target gene were generated using an artificial gene synthesis service (FASMAC, Kanagawa, Japan) (Figure 1). The DNA fragment was double-digested with *Xho*I and *Bam*HI and ligated to pEALSR2L5R, which had been digested with the same enzymes (Yamagishi and Yoshikawa 2013).

### Preparation of viral RNA

Viral RNA was prepared as previously described (Li et al. 2004). Briefly, pCALSR1, pCALSR2, ALSV-DsCHS, ALSV-DsACS1, ALSV-DsACO1A and ALSV-DsACO1B plasmids were independently introduced into *A. tumefaciens* strain GV3101::pMP90. *Nicotiana benthamiana* leaves were inoculated with a 1 : 1 mixture of *Agrobacterium* cultures transformed with pCALSR1 and pCALSR2 (or ALSV-DsCHS, ALSV-DsACS1, ALSV-DsACO1A and ALSV-DsACO1B). One month after inoculation, the upper leaves were tested for viral infection using RT-PCR. Infected leaves were ground with buffer and the solution, then rub-inoculated onto *N. benthamiana* leaves. ALSV was extracted from infected *N. benthamiana* leaves with extraction buffer (0.1 M Tris-HCl, pH 7.8, 0.1 M NaCl, and 5 mM MgCl_2_) and concentrated with bentonite solution. Viral RNA was obtained via phenol–chloroform (1 : 1) extraction from the solution followed by precipitation with ethanol. RNA concentration was measured by NanoDrop, and stored at −80°C.

### Inoculation of viral RNA to plants

**Figure 2:**
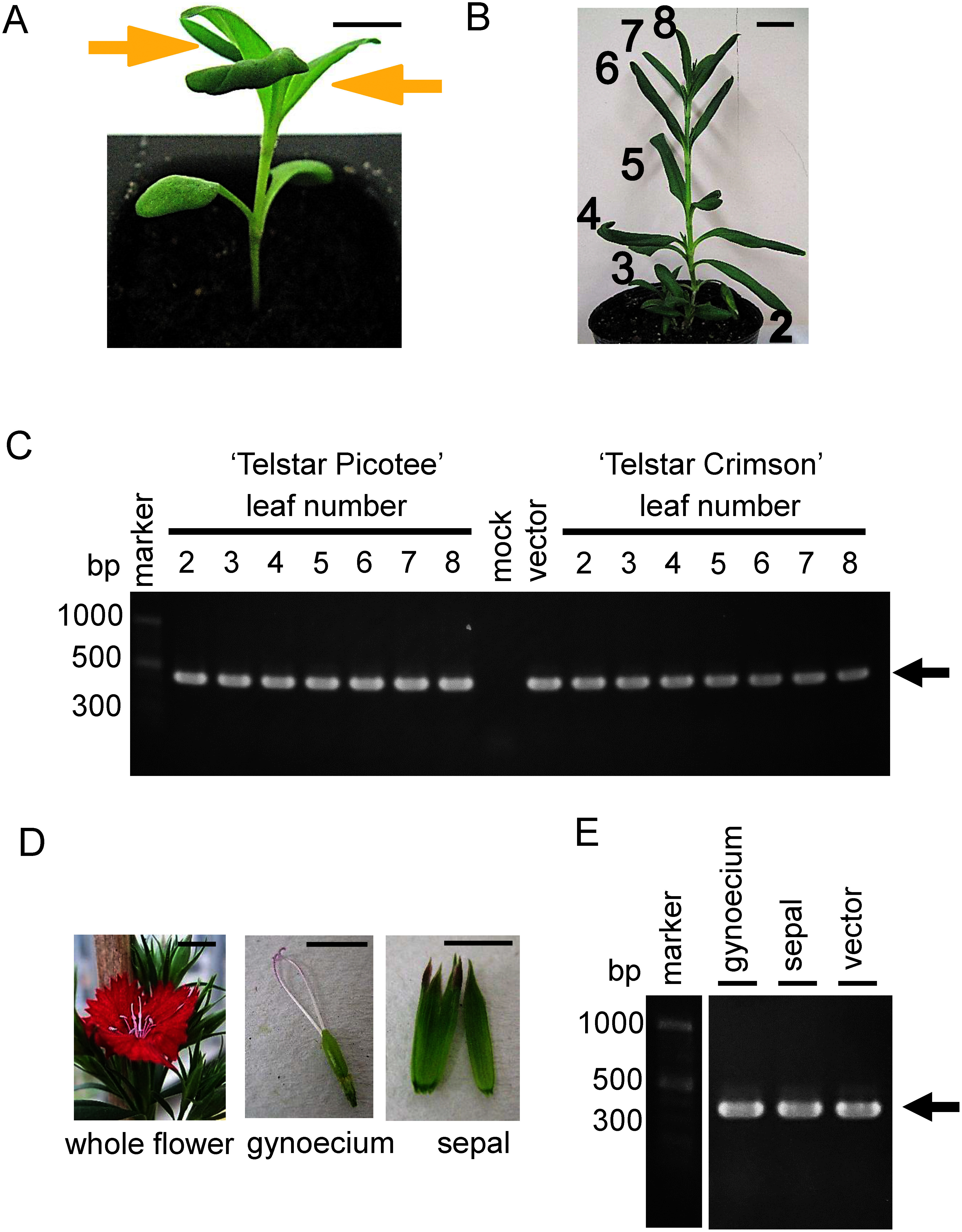
Figure 2. Infection of *Dianthus* plants by wtALSV. (A) *Dianthus* seedling grown on soil. Yellow arrowheads indicated expanded true leaves in which gold particles were bombarded. Bar=10 mm. (B) wtALSV-infected plants at 30 days after post inoculation. Number of true leaves 2 to 8 were harvested and used for RNA extraction. Bar=20 mm. (C) RT-PCR assay of wtALSV infection to leaves of *Dianthus* ‘Telstar Picotee’ and ‘Telstar Crimson’. The 2 to 8 indicated the number of true leaves. An arrow indicates DNA products (360 bp). (D) Whole flower, gynoecium and sepal of ‘Telstar Crimson’ plants. Bars=10 mm. (E) RT-PCR assay of wtALSV infection to flower organs of ‘Telstar Crimson’ plants.

**Figure 3:**
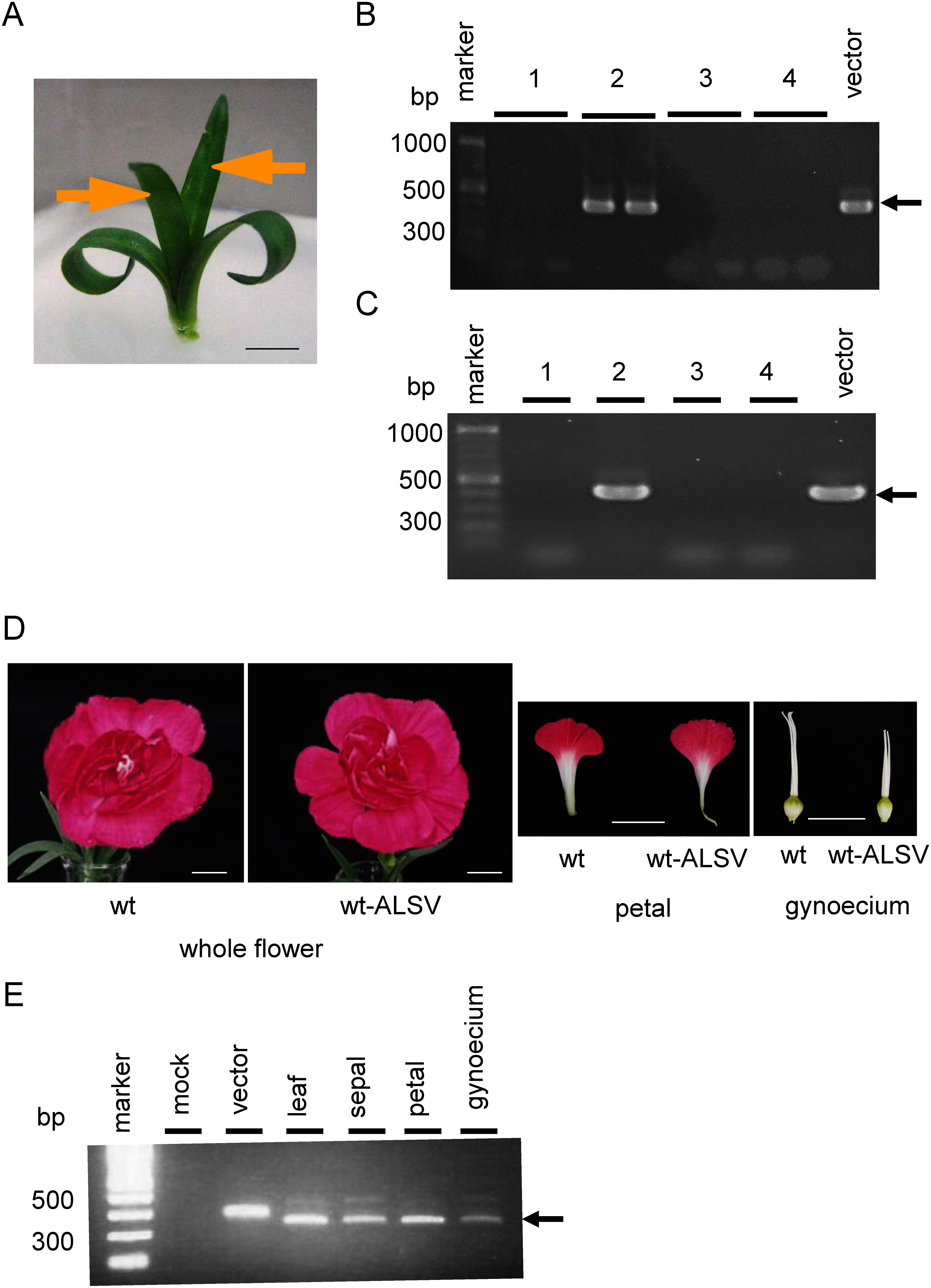
Figure 3. Infection of carnation plants by wtALSV. (A) Carnation grown aseptically on MS medium. Yellow arrows indicated expanded true leaves in which gold particles were bombarded. Bar=10 mm. (B) RT-PCR assay of wtALSV infection to inoculation leaves of carnation ‘Ariel’ at 30 days after post inoculation. Numbers 1 through 4 indicated independent plants. An arrow indicates DNA products (400 bp). (C) RT-PCR assay of wtALSV infection to upper leaves of carnation ‘Ariel’ at 30 days after post inoculation. Numbers 1 through 4 indicated independent plants. (D) The whole flower, gynoecium, and petal of non-infected and wt-ALSV-infected ‘Ariel’ plants. Bars=10 mm. (E) RT-PCR assay of wtALSV infection to flower organs of ‘Ariel’ plants.

Gold particles (0.6 µm) (Bio-Rad Laboratories, CA, USA) were covered with viral RNA, as described previously (Li et al. 2019). For each bombardment of wild-type ALSV (wtALSV), 7 µg of RNA was used, whereas 10 µg of RNA was used for the bombardment of ALSV-DsCHS1. In the *Dianthus* cultivars, the two expanded true leaves were inoculated by bombardment with RNA-coated gold particles using a GDS-80 gene gun system (NepaGene, Ichikawa, Japan) (Figure 2A). In the carnation, the two expanded true leaves, which were the tips of the cultured plants, were inoculated by bombardment (Figure 3A). A shot of gold particles was applied to each leaf at a pressure of 20 psi.

For checking infection, first strand cDNA was synthesized using 1 µg RNA, oligo (dT) primer, and PrimeScriptII 1st Strand cDNA Synthesis Kit (TaKaRa Bio Inc.) following the manufacturer’s instructions. PCR amplification was performed using 1 µl of template cDNAs and following primers: ALSR2fw1 and ALSR2rv1 for carnation and ALSR2fw2 and ALSR2rv2 for *Dianthus*. The PCR amplification was conducted with 30 cycles at 94°C for 30 s, 58°C for 20 s, 72°C for 1 min after an initial incubation at 95°C for 1 min. The PCR products were then electrophoresed in a 2% agarose gel. Infection rate was calculated as Infected plants/inoculated plants (%).

### Gene expression analysis

Expression analyses of the *DsCHS1* in infected *Dianthus* petals were carried out using semiquantitative reverse transcriptase-PCR (RT-PCR) using a forward primer (DsCHS1fw) and a reverse primer (DsCHS1rv) (Supplementary Table S1). PCR was performed using Ex Taq DNA polymerase (TaKaRa Bio Inc.) under the following conditions: 30 s at 95°C, 27 cycles of 5 s at 95°C, and 60 s at 68°C. Expression analyses of *DsACS1* and *DsACO1* in infected *Dianthus* petals were carried out via quantitative real-time RT-PCR (qRT-PCR). *DsACS1* (AB543544) was analyzed using the DsACS1fw and DsACS1rv. *DsACO1* (LC880074) was analyzed using the DsACO1fw and DsACO1rv. The qRT-PCR was performed using SYBR Premix Ex Taq (TaKaRa Bio Inc.) and signals were detected using the Thermal Cycler Dice Real Time System TP800 (TaKaRa Bio Inc.) following the manufacturer’s instructions. The PCR conditions were as the follows: 30 s at 95°C, 30 cycles of 5 s at 95°C, and 30 s at 68°C. The *Dianthus ubiquitin* (*DsUbq*) gene was used as a control for expression analysis using the qRT-PCR (Nomura et al. 2012).

## Results

### Infection of wild-type ALSV

We investigated whether wtALSV could infect *Dianthus* and carnation plants. Seedlings of two cultivars of *Dianthus*, ‘Telstar Picotee’ and ‘Telstar Crimson’ were inoculated with the wtALSV via particle bombardment (Figure 2A). The infection rates of ‘Telstar Picotee’ and ‘Telstar Crimson’ were 35% and 56%, respectively (Table 1). wtALSV infection was observed in all upper leaves (Figure 2B, C) and in the gynoecium and sepals of flowers (Figure 2D, E). Infected plants did not show significant changes in growth or symptoms of viral disease compared to uninfected plants.

**Table 1:** Table 1. Infection rates of ALSV vectors to seedling (*D. hybrida*) and in vitro culture plants (*D. caryophyllus* L.).

Vectors	Species	Cultivar	Infected plants/Total inoculated plants (%)
wt-ALSV	*D. hybrida*	‘Telstar Picotee’	9/26 (35)
wt-ALSV	*D. hybrida*	‘Telstar Crimson’	15/27 (56)
wt-ALSV	*D. caryophyllus* L.	‘Ariel’	8/25 (32)
ALSV-DsCHS	*D. hybrida*	‘Telstar Picotee’	6/20 (30)
ALSV-DsACS1	*D. hybrida*	‘Telstar Picotee’, ‘Telstar Crimson’	7/32 (22)
ALSV-DsACO1A	*D. hybrida*	‘Telstar Crimson’	12/15 (80)
ALSV-DsACO1B	*D. hybrida*	‘Telstar Crimson’	9/15 (60)

Carnation varieties are not seed-propagated, instead proliferating via vegetative propagation. Therefore, carnation plants grown aseptically on MS medium were used for inoculation with ALSV vectors (Figure 3A). The infection efficiency was 32% (Table 1) and infection was observed in the inoculated and upper leaves (Figure 3B, C). Infection with wtALSV was also observed in the sepals, petals, and gynoecium of flowers (Figure 3D, E). Infected plants did not show growth changes or symptoms of viral disease in aseptic and pot cultures compared to uninfected plants.

### Infection of ALSV-DsCHS

To investigate whether ALSV could be used for VIGS of the petals of *Dianthus* plants, we studied the modification of color in the petals of *Dianthus*. The cDNA clone of *DsCHS* was isolated from *Dianthus* ‘Telstar Picotee’ using cloning primers. By comparing the obtained sequences, nucleotide sequences of *DsCHS* showed 99% identity to those of *chalcone synthase* (*CHS*) genes from carnation (*Dianthus caryophyllus*), *Dianthus chinennsis*, and *Dianthus monspessulanus*, which have been registered with the following accession numbers: AF267173, LC377191, KX893854, and AF267173, respectively (Morimoto et al. 2019; Ogata et al. 2004).

**Figure 4:**
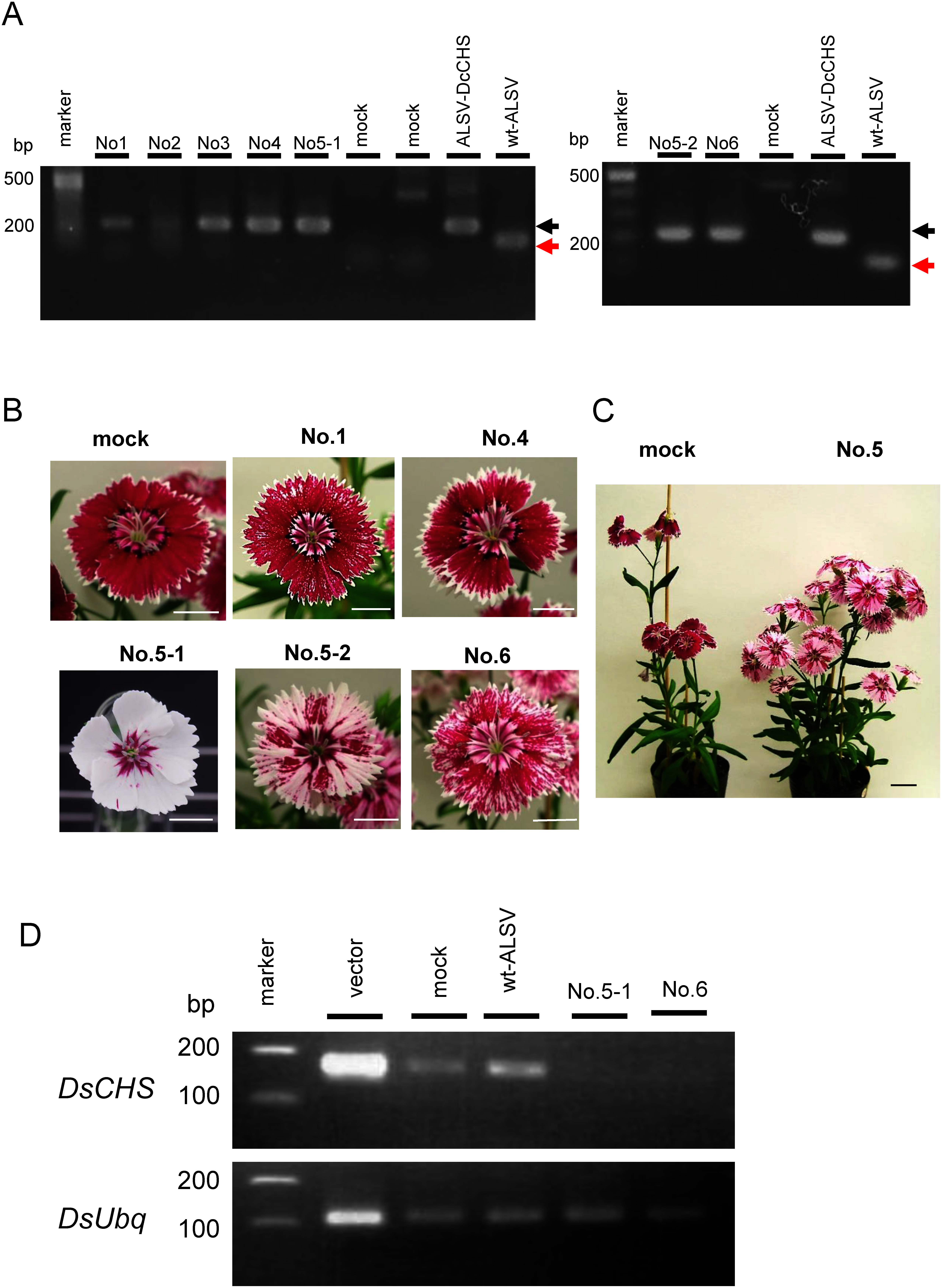
Figure 4. Infection of *Dianthus* plants by ALSV-DsCHS. (A) RT-PCR assay of DsCHS-ALSV infection to leaves of *Dianthus* ‘Telstar Picotee’ at 30 days after post-inoculation. The No. 1 through No. 6 indicated independent plants. Black arrows indicated the 250‑bp DNA product containing the target gene fragment, whereas red arrows indicated the 49‑bp DNA fragment lacking the target gene. (B) Flowers of ‘Telstar Picotee’ plants infected by DsCHS-ALSV at 60 days after post inoculation. The No. 5-1 and No. 5-2 were flowers obtained from different branches of the same plant infected by DsCHS-ALSV. Bars=10 mm. (C) Whole plant of No. 5 infected by DsCHS-ALSV. Bar=20 mm. (D) Semi-quantitative RT-PCR of the *DsCHS* in petals of *Dianthus* ‘Telstar Picotee’ infected with DsCHS-ALSV at 60 days post inoculation. PCR for *DsCHS* was performed for 27 cycles, and PCR for *DsUbq* was performed for 25 cycles.

A 300 bp fragment of *DsCHS* was synthesized artificially and cloned into the sites between *Xho*I and *BamH*I of the RNA2 vector of ALSV (Figure 1B). The recombinant virus was designated ALSV-DsCHS and used for further experiments. RNA preparation from the leaves of *Dianthus* infected with ALSV-DsCHS was bombarded onto the true leaves of seedlings in the ‘Telstar Picotee’. Of the 20 plants, 6 (30%) were infected with ALSV-DsCHS (Figure 4A). After flowering, infected plants with large and small white sectors on the petals were observed (Figure 4B). The plant No. 5 exhibited the largest area of white sector petals, with some petals almost completely white. Petals with white sectors were perceived in all flowers of this plant (Figure 4C) and persisted at least one month after inoculation. For semi-quantitative RT-PCR analysis, total RNA was extracted from the white sectors of plants No. 5 and No. 6. Transcript of *DsCHS* was not detected in the white petals (Figure 4D). These results suggest that the ALSV vector can be used for gene function analysis of petals in *Dianthus*.

### Infection of ALSV-DsACS1, ALSV-DsACO1A and ALSV-DsACO1B

**Figure 5:**
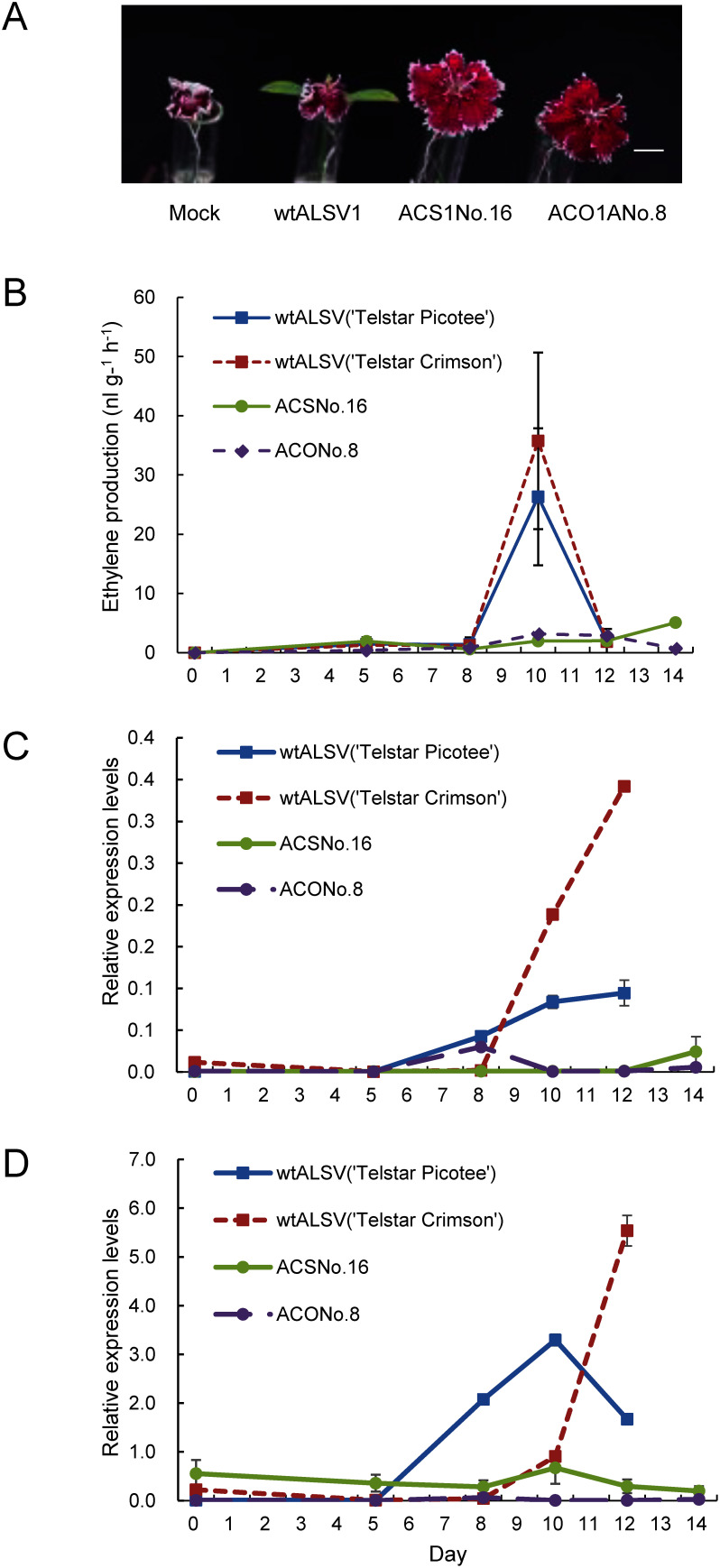
Figure 5. Flower longevity of *Dianthus* infected by ALSV-DsACS1 and ALSV-DsACO1A. (A) Flowers of ACS1 No. 16 and ACO1A No. 8 at 12 days after harvested from plants infected with ALSV-DsACS1 and ALSV-DsACO1A, respectively. Flowers were harvested at the fully open petal stage. Bar=10 mm. (B) Ethylene production from flowers of plants infected with ACS1 No. 16, ACO1A No. 8, and wtALSV. (C) Quantitative RT-PCR assay of *DsACS1* in the gynoecium of plants infected with ACS1 No. 16, ACO1A No. 8, and wtALSV. All expression levels wer normalized to the level of *DsUbq3*. (D) Quantitative RT-PCR assay of *DsACO1* in the gynoecium of plants infected with ACS1 No. 16, ACO1A No. 8, and wtALSV. All expression levels wer normalized to the level of *DsUbq3*.

We further confirmed whether the ALSV system could alter flower longevity to determine whether it could be used to analyze the functions of genes other than *CHS* in *Dianthus*. In addition, we confirmed whether gene silencing by the ALSV system works in gynoecia as well as in the petals. The mechanism of flower senescence in *Dianthus* is a typical ethylene-dependent regulation of the ethylene biosynthetic genes *ACS* and *ACO* (Satoh et al. 2000). A 201 bp fragment of *DsACS1* and two 201 bp fragments of *DsACO1* (*DsACO1A* and *DsACO1B*) were synthesized artificially and cloned into the sites between *Xho*I and *BamH*I of the RNA2 vector of ALSV (Figure 1B). These recombinant viruses were designated ALSV-DsACS1, ALSV-DsACO1A, and ALSV-DsACO1B and were used for further experiments. The infection rates of ALSV-DsACS1, ALSV-DsACO1A, and ALSV-DsACO1B were 22, 80, and 60%, respectively (Table 1). Plants infected with each recombinant virus were grown in a greenhouse and examined for flowering longevity. Supplementary Tables S2 and S3 show the longevity of potted and cut flowers, respectively. Several strains showed prolonged flower retention in potted flowers compared with plants infected with wtALSV (Figure 5A). Flowers with extended longevity in potted plants also exhibited extended longevity in the cut flowers. The maximum duration of flower longevity was extended by 8.6 days in potted flowers and 8.0 days in cut flowers. Two recombinant lines with extended flowering times were selected for ethylene production and the expression of ethylene biosynthetic genes in cut flowers. The wtALSV-infected plants reached their peak ethylene production 10 days after flowering (Figure 5B). However, plants infected with ALSV-DsACS1 or ALSV-DsACO1A exhibited low ethylene production. The expression levels of *DsACS1* and *DsACO1* in recombinant virus-infected plants were also examined (Figure 5C, D). The wtALSV-infected plants showed an increase in the expression of *DsACS1* 10 days after flowering, whereas the expression of *DsACS1* remained low in plants infected with ALSV-DsACS1 and ALSV-DsACO1A. The expression of *DsACO1* increased in wt-ALSV-infected plants after 10 days of flowering, whereas the expression of *DsACO1* was slightly upregulated in plants infected with ALSV-DsACS1 but remained low in plants infected with ALSV-DsACO1A.

## Discussion

The ALSV vector system is a powerful tool for studying the functions of genes of interest in a broad range of plant species according to their VIGS inducers. In carnations, *Agrobacterium*-mediated transformation has been reported (Nontaswatsri et al. 2004; van Altvorst et al. 1995). For example, transgenic carnations created via introducing the *F3′ 5′H* gene, a key gene for the synthesis of the delphinidin, exhibit blue flower petals (Fukui et al. 2003; Tanaka et al. 1998). However, while genetically modified blue carnations have been successfully commercialized worldwide (Tanaka and Brugliera 2013), the efficiency of transformation varies depending on the cultivar and gene transfer rates are often low (Nontaswatsri et al. 2004; van Altvorst et al. 1995). Additionally, obtaining transgenic carnation plants is time-consuming. Transformation with viral vectors is expected to reduce the time required to obtain transgenic plants significantly.

In this study, using particle bombardment, wtALSV infected soil-grown plants of two *Dianthus* cultivars, ‘Telstar Picotee’ and ‘Telstar Crimson’ but failed to infect soil-grown carnation cultivar ‘Ariel’. wtALSV successfully infected in vitro-grown carnation plants. Differences in infection rates between soil- and in vitro-grown plants have been observed in strawberries (Li et al. 2019). These results suggest that the infection efficiency of ALSV vectors depends on the environmental conditions under which the plants are grown and that in vitro plants may be more susceptible to infection by ALSV vectors. The ALSV vector may be a useful viral vector by optimizing plant growth conditions in many plant species.

Using ALSV-DsCHS, some *Dianthus* plants were phenotyped as *DsCHS* gene-silenced plants, exhibiting petals with white areas (Figure 4B, C). These results indicate that the ALSV vector is advantageous for gene function analysis using VIGS in *Dianthus*. The varying extent of the white area in the petals between ALSV-DsCHS-infected plants indicates that the level of silencing of *DsCHS* expression differed. Silencing of *DsCHS* by VIGS may be affected by environmental conditions as high temperatures suppress the proliferation of ALSV (Yamagishi et al. 2016). ALSV-infected apple and pear plants showed no movement of ALSV into new growing tissues after 2 weeks of treatment at 37°C. In *Petunia*, the location and amount of gene silencing in a plant can be influenced by environmental factors and inoculation methods in the case of virus-induced gene silencing (Broderick and Jones 2014; Chen et al. 2004; Spitzer et al. 2007). Optimization of VIGS technology and environmental factors using TRV vectors has significantly increased the efficiency of the silencing phenotype in both flowers and leaves of *Petunia* (Broderick and Jones 2014). Thus, the optimization of environmental factors when using ALSV vectors will also increase the efficiency of the silenced phenotype in *Dianthus*.

During senescence, *Dianthus* flowers produce ethylene, which is accompanied by increased expression of *DsACS1* and *DsACO1* (Figure 5). However, the expression of these genes did not increase in plants infected with ALSV-DsACS1 or ALSV-DsACO1A because of the suppression of autocatalytic ethylene production. In previous studies, ethylene production increased autocatalytically during flower senescence in carnations, accompanied by increases in *DsACS1* and *DsACO1* (Kosugi et al. 2002; Satoh et al. 2000; Xu et al. 2021). Exogenous applied ethylene induces the expression of *DcACS1* and *DcACO1* in a manner similar to that of flower senescence (Woodson et al. 1992). In addition, the increase of *DcACS1* and *DcACO1* transcripts in senesceing flowers is prevented by treatment with ethylene inhibitor. These findings suggest that ethylene biosynthesis genes is under positive feedback regulation during flower senescence. Although petals are major contributors to ethylene production during senescence in *Dianthus* flowers, they produce significant amounts of ethylene prior to the onset of ethylene production from petals (Harada et al. 2011; Nichols 1977; ten Have and Woltering 1997; Woodson et al. 1992). In the present study, ethylene production and ethylene biosynthesis genes in *Dianthus* plants infected with ALSV-DsACS1, ALSV-DsACO1A, and ALSV-DsACO1B were repressed, resulting in prolonged flower longevity. These results suggest that the ALSV system functions in the gynoecium and petals of *Dianthus* plants.

In previous studies, *Agrobacterium*-mediated transformation yielded transgenic carnation plants that contained transgenes, such as *DcACS1* and *DcACO1*, and the mutant of the ethylene receptor, *etr1-1*. The findings reported lower transformation efficiencies of *DcACS1* (0.3 to 0.5%) than of *DcACO1* (Bovy et al. 1999; Iwazaki et al. 2004; Kinouchi et al. 2006; Savin et al. 1995). These findings suggest that *ACS* may have a negative effect on *Agrobacterium*-based transformations. Therefore, ALSV may be effective for transformation with transgenes that are detrimental to *Agrobacterium*-mediated transformation. Interestingly, the plant hormone ethylene affects the efficiency of *Agrobacterium*-mediated transformations in some plants (Nonaka and Ezura 2014). Transformation efficiency in some plant species increased when ethylene inhibitors, such as aminoethoxyvinylglycine and silver ions, were added to the tissue culture media and increased in mutant ethylene-receptor plants with reduced ethylene sensitivity (Ezura et al. 2000; Nonaka et al. 2008). In contrast, increasing ethylene production by adding an ethylene precursor, 1-aminocyclopropane-1-carboxylic acid, suppresses gene transfer (Davis et al. 1992; Ezura et al. 2000). It would be interesting to determine whether the use of ethylene inhibitors improves the transformation efficiency when the *DcACS1* gene is transduced into carnation plants.

Although direct comparisons between methods of genetic transformation are challenging, the virus vector-mediated transformation method offers advantages in terms of the efficiency and speed of producing transgenic plants compared to *Agrobacterium*-mediated transformation. Therefore, ALSV is a useful tool for the functional analysis of endogenous genes in *Dianthus* plants. Comprehensive analyses of the whole genome and expressed genes of the carnation have been conducted and sequence information on most of the endogenous genes is available (Tanase et al. 2012; Yagi et al. 2014). In the future, ALSV could facilitate the analysis of genes involved in phenomena specific to *Dianthus*, enabling the production of plants with improved characteristics.

## Data Availability

The data that support the findings of this study are available from the corresponding author, K.T., upon reasonable request.

## Description of supplementary files

Supplementary Table S1. List of primers and VIGS target sequence of CHS1, ACS1, and ACO1A, B.

Supplementary Table S2. Flower longevity of potted *Dianthus* plants which were infected by ALSV, ALSV-ACS1, ALSVACO1A and ALSV-ACO1B.

Supplementary Table S3. Flower longevity of cut *Dianthus* flowers which were infected by ALSV, ALSV-ACS1, ALSVACO1A and ALSV-ACO1B.
