# Supplementary material for: Virus-induced gene silencing in Dianthus by an apple latent spherical virus vector system
Source: Plant Biotechnol (Tokyo). 2026 Mar 25;43(1):17–25. doi: 10.5511/plantbiotechnology.25.0922a (PMC13170821; doi:10.5511/plantbiotechnology.25.0922a)
Supplement: Supplementary Data [file plantbiotechnology-43-1-25.0922a-s001.pdf]

Supplementary Table S1 List of primers and VIGS target sequence of CHS1, ACS1, and ACO1A,B

Target gene	Name	Sequence(5' - 3')	Application
DsCHS1	DsCHSFullfw	ATGATGAATAGTGACAACAACACTAGT	cloning
	DsCHSFullrv	TTAAAGACTACGCATAAGAA	
ALSVR2	ALSR2fw1	TGTCCTCAAGAGGCCAAACT	checking infection for carnation
	ALSR2rv1	CCTCCCTGGTAAATTCTGGA	
ALSVR2	ALSR2fw2	GCGAGGCACTCCTTA	checking infection forDianthus
	ALSR2rv2	GCAAGGTGGTCGTGA	
DsCHS1	DsCHS1fw	ACATGCCAGGAGCCGACTAC	qPCR
	DsCHS1rv	ACAGACAACAAGAACGCGCG	
DsACS1	DsACS1fw	GATGCCTCCAAGGAAACGCG	qPCR
	DsACS1rv	CTCTCCCTCGACCGTTGCTT	
DsACO1	DsACO1fw	TCCGAGCTCCCTGATCTCGA	qPCR
	DsACO1rv	CCATTGGCACCATGGAAGGC	
DsUbq3	DsUbq3fw	GTTGTTGGTTTCAGGGCTGGTTTG	qPCR
	DsUbq3rv	CTACGGTAATTGAGAATTCACACCGAAATG	
DsCHS1	DsCHS1	CGGAGGTTCCTAAGCTAGGCAAAGAGGCCGCTGTTAA GGCCATTAAGGAGTGGGGCCAACCTAAGTCCAAAATC ACTCACGTCATCTTTTGCACTACCTCCGGTGTCGACATG CCAGGAGCCGACTACCAGCTCACCAAGCTCCTCGGGTT ATGTCCCTCTGTCCGCCGCTTCATGCTCTACCAACAGG GTTGCTATGCTA	The 201-nt regions for VIGS target
DsACS1	DsACS1	AATGCGAAAAATATACACCTTGTGTGTGACGAGATATAT GCAACCACAGTATTTAATTCGCCGAGCTTTATAAGTGTT GCTGAGGTTATAAAGGACATGCCTCATGTAAATCAAGA CCTTGTTCATATTTATATAGTTTGTCCAAGGACATGGG CATGCCCGGCTTTAGGGTTGGGATCATTTACTCTTATAA TGACCGT	The 201-nt regions for VIGS target
DsACO1	DsACO1A	CTTGAGGAGCTGGAGGCCGCTGACCTTGTCGTCCTGG AACAAGAGAATGATGCCACCAGCGTCGGTGTGGGCCC TAAGTCCTTTGATAAGGTCGGGTTTGGGGCAAGGCGGG TAGTTGCTGACCTTGGTACCAAAAGTGGGGCCATTGGC ACCATAGAAGGCATTCTTAAGGTAGCCTTTCTCAAGGC CAAGGTTCTCACA	The 201-nt regions for VIGS target
DsACO1	DsACO1B	CAACTTGAGGTTATTACAAATGGCAAGTACAAGAGTGT GATGCACCGCGTGATAGCGCAGACAGATGGTAACAGG ATGTCGATAGCATCATTCTACAACCCGGGAAGTGATGC CGTGATTTACCCGGCGCCAACATTGGTGGAAAAAGAA GAGGAGAAATGCAGAGCATACCCAAAATTTGTGTTCGA GGATTACATGAAT	The 201-nt regions for VIGS target

Supplementary Table S2 Flower longevity of potted Dianthus plants which were infected by ALSV, ALSV-ACS1, ALSVACO1A and ALSV-ACO1B.

Vector	Cultivar	Line No	Flower longevity (day)
wt-ALSV	'Telstar Picotee'	1	12.3 ± 0.5
wt-ALSV	'Telstar Picotee'	2	12.5 ± 0.6
wt-ALSV	'Telstar Picotee'	3	13.7 ± 0.3
wt-ALSV	'Telstar Crimson'	1	10.6 ± 0.4
wt-ALSV	'Telstar Crimson'	2	10.3 ± 0.6
wt-ALSV	'Telstar Crimson'	3	9.5 ± 0.5
ALSV-DcACS1	'Telstar Picotee'	2	16.7 ± 0.4*
ALSV-DcACS1	'Telstar Picotee'	3	14.8 ± 0.7
ALSV-DcACS1	'Telstar Picotee'	4	17.1 ± 0.8*
ALSV-DcACS1	'Telstar Picotee'	16	18.6 ± 0.7*
ALSV-DcACS1	'Telstar Picotee'	18	16.6 ± 0.6*
ALSV-DcACS1	'Telstar Crimson'	3	13.7 ± 1.0
ALSV-DcACS1	'Telstar Crimson'	8	14.4 ± 0.7
ALSV-DcACO1A	'Telstar Crimson'	2	11.6 ± 0.5
ALSV-DcACO1A	'Telstar Crimson'	3	15.9 ± 0.7*
ALSV-DcACO1A	'Telstar Crimson'	5	13.5 ± 0.2*
ALSV-DcACO1A	'Telstar Crimson'	6	13.4 ± 0.3
ALSV-DcACO1A	'Telstar Crimson'	7	16.3 ± 0.6*
ALSV-DcACO1A	'Telstar Crimson'	8	15.8 ± 0.4*
ALSV-DcACO1A	'Telstar Crimson'	9	15.3 ± 0.6*
ALSV-DcACO1A	'Telstar Crimson'	10	9.6 ± 0.3
ALSV-DcACO1A	'Telstar Crimson'	11	14.6 ± 0.4*
ALSV-DcACO1A	'Telstar Crimson'	12	16.3 ± 0.1*
ALSV-DcACO1A	'Telstar Crimson'	13	14.4 ± 0.2*
ALSV-DcACO1A	'Telstar Crimson'	14	11.5 ± 0.6
ALSV-DcACO1B	'Telstar Crimson'	1	17.9 ± 1.0*
ALSV-DcACO1B	'Telstar Crimson'	2	15.4 ± 1.1
ALSV-DcACO1B	'Telstar Crimson'	3	16.2 ± 0.8*
ALSV-DcACO1B	'Telstar Crimson'	4	13.9 ± 1.0
ALSV-DcACO1B	'Telstar Crimson'	5	15.3 ± 0.8*
ALSV-DcACO1B	'Telstar Crimson'	6	12.1 ± 0.2
ALSV-DcACO1B	'Telstar Crimson'	7	13.9 ± 0.5
ALSV-DcACO1B	'Telstar Crimson'	8	15.1 ± 1.1*
ALSV-DcACO1B	'Telstar Crimson'	9	14.4 ± 1.5

Asterisks indicate the level of significance based on a two-sample t -test at 5%.

Supplementary Table S3 Flower longevity of cut Dianthus flowers which were infected by ALSV, ALSV-ACS1, ALSVACO1A and ALSV-ACO1B.

Vector	Cultivar	Line No	Flower longevity (day)
wt-ALSV	'Telstar Picotee'	1	10.0 ± 0.5
wt-ALSV	'Telstar Picotee'	2	11.0 ± 0.7
wt-ALSV	'Telstar Picotee'	3	12.6 ± 1.5
wt-ALSV	'Telstar Crimson'	1	9.5 ± 1.1
wt-ALSV	'Telstar Crimson'	2	11.0 ± 1.2
wt-ALSV	'Telstar Crimson'	3	8.2 ± 1.1
ALSV-DcACS1	'Telstar Picotee'	2	13.8 ± 1.2*
ALSV-DcACS1	'Telstar Picotee'	3	14.2 ± 1.4
ALSV-DcACS1	'Telstar Picotee'	4	15.8 ± 1.6*
ALSV-DcACS1	'Telstar Picotee'	16	15.5 ± 1.3*
ALSV-DcACS1	'Telstar Picotee'	18	16.0 ± 1.5*
ALSV-DcACS1	'Telstar Crimson'	3	12.8 ± 1.2
ALSV-DcACS1	'Telstar Crimson'	8	12.1 ± 1.0
ALSV-DcACO1A	'Telstar Crimson'	2	12.5 ± 1.3
ALSV-DcACO1A	'Telstar Crimson'	3	15.0 ± 1.6*
ALSV-DcACO1A	'Telstar Crimson'	5	12.3 ± 1.6
ALSV-DcACO1A	'Telstar Crimson'	6	14.2 ± 1.4*
ALSV-DcACO1A	'Telstar Crimson'	7	13.6 ± 1.5*
ALSV-DcACO1A	'Telstar Crimson'	8	16.7 ± 1.7*
ALSV-DcACO1A	'Telstar Crimson'	9	14.5 ± 1.4*
ALSV-DcACO1A	'Telstar Crimson'	10	13.0 ± 1.6*
ALSV-DcACO1A	'Telstar Crimson'	11	13.7 ± 1.4*
ALSV-DcACO1A	'Telstar Crimson'	12	13.6 ± 1.5*
ALSV-DcACO1A	'Telstar Crimson'	13	15.1 ± 1.7*
ALSV-DcACO1A	'Telstar Crimson'	14	14.5 ± 1.4*
ALSV-DcACO1B	'Telstar Crimson'	1	18.0 ± 2.1*
ALSV-DcACO1B	'Telstar Crimson'	2	13.3 ± 1.4*
ALSV-DcACO1B	'Telstar Crimson'	3	13.6 ± 1.6*
ALSV-DcACO1B	'Telstar Crimson'	4	12.8 ± 1.4
ALSV-DcACO1B	'Telstar Crimson'	5	13.0 ± 1.5
ALSV-DcACO1B	'Telstar Crimson'	6	8.5 ± 1.0
ALSV-DcACO1B	'Telstar Crimson'	7	13.7 ± 1.7
ALSV-DcACO1B	'Telstar Crimson'	8	14.5 ± 1.9*
ALSV-DcACO1B	'Telstar Crimson'	9	13.5 ± 1.5

Asterisks indicate the level of significance based on a two-sample t -test at 5%.
